# Campylobacteriosis, Salmonellosis, Yersiniosis, and Listeriosis as Zoonotic Foodborne Diseases: A Review

**DOI:** 10.3390/ijerph15050863

**Published:** 2018-04-26

**Authors:** Agnieszka Chlebicz, Katarzyna Śliżewska

**Affiliations:** Institute of Fermentation Technology and Microbiology, Department of Biotechnology and Food Sciences, Lodz University of Technology, Wólczańska 171/173, 90-924 Łódź, Poland

**Keywords:** zoonoses, bacterial pathogens, foodborne diseases

## Abstract

Zoonoses are diseases transmitted from animals to humans, posing a great threat to the health and life of people all over the world. According to WHO estimations, 600 million cases of diseases caused by contaminated food were noted in 2010, including almost 350 million caused by pathogenic bacteria. *Campylobacter*, *Salmonella*, as well as *Yersinia enterocolitica* and *Listeria monocytogenes* may dwell in livestock (poultry, cattle, and swine) but are also found in wild animals, pets, fish, and rodents. Animals, often being asymptomatic carriers of pathogens, excrete them with faeces, thus delivering them to the environment. Therefore, pathogens may invade new individuals, as well as reside on vegetables and fruits. Pathogenic bacteria also penetrate food production areas and may remain there in the form of a biofilm covering the surfaces of machines and equipment. A common occurrence of microbes in food products, as well as their improper or careless processing, leads to common poisonings. Symptoms of foodborne infections may be mild, sometimes flu-like, but they also may be accompanied by severe complications, some even fatal. The aim of the paper is to summarize and provide information on campylobacteriosis, salmonellosis, yersiniosis, and listeriosis and the aetiological factors of those diseases, along with the general characteristics of pathogens, virulence factors, and reservoirs.

## 1. Introduction

Zoonoses, or diseases of animal origin, are defined as diseases transmitted between animals and humans as a consequence of a direct contact, indirect environmental contact, or through food [1]. Among recognised pathogens causing human diseases, almost 60% are of animal origin. They cause such diseases as toxoplasmosis, anthrax, rabies, Ebola haemorrhagic fever, severe acute respiratory syndrome (SARS), and primary HIV infection [2,3].

Already in 1906, doctor Silvio J. Bonansea described in his paper titled “Veterinary Hygiene Applied to the Protection of Man against Zoonoses” how animal health and hygiene are important for the production of safe and healthy meat and milk [4]. Almost 50 years later, in November 1950 in Geneva, during the first meeting of the Expert Group on Zoonoses formed by the World Health Organisation (WHO) and the United Nations Food and Agriculture Organisation (FAO), a list of 86 diseases transmitted from animals to humans was identified. Twenty of those diseases were caused by bacteria [5]. Nowadays, it is estimated that among 1400 pathogens causing human diseases, 800 are of animal origin [6].

**Figure 1:**
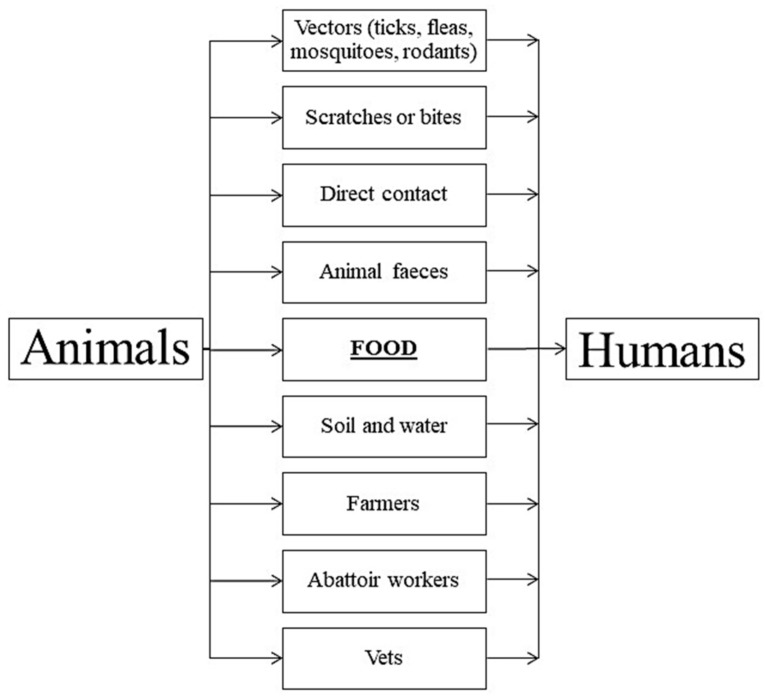
Routes of diseases transmission from animals to humans [7,8].

There are numerous mechanisms of the transmission of zoonoses, and some diseases are transmitted in various ways, which significantly hinders the diagnostic process (Figure 1) [7].

Symptoms of food poisoning may be variable, ranging from mild and transient, including nausea, vomiting, and malaise, to life-threatening kidney and liver failure, paralysis, and the dysfunction of the nervous system and brain. Cases caused by consumption of unsafe and contaminated food may also account for some instances of early death. Their global count may reach 4 million a year [9,10].

**Figure 2:**
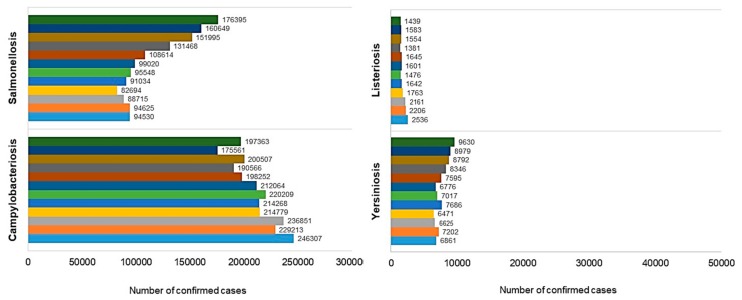
Number of confirmed cases of selected bacterial zoonoses in the European Union between 2005–2016 [1,13,16,17,18,19,20,21,22,23,24,25].

According to the WHO report published in 2015, almost 600 million cases of diseases caused by contaminated food were noted in 2010, including almost 350 million caused by pathogenic bacteria [10]. Bacterial diseases of animal origin, e.g., caused by *Campylobacter* sp., *Salmonella* sp., *Listeria* sp., or the *Enterobacteriaceae* family, constitute a serious health risk both in developing countries and in advanced ones as well, such as EU countries and the United States. It is estimated that in the US, the number of food poisonings may reach as high as 48 million cases a year, with salmonellosis and campylobacteriosis alone affecting as many as 2 million people a year [11,12]. In the EU, there are over 200,000 cases of bacterial zoonoses noted annually with presumably much higher numbers of real cases. According to the 2017 report of the European Food Safety Authority (EFSA) and the European Centre for Disease Prevention and Control (ECDC), the most common causes of food-borne zoonotic diseases were *Campylobacter* and *Salmonella* bacteria (Figure 2) [13]. In Canada, the annual incidence of food poisonings ranges between 3.1 and 5.0 million. In Australia, the incidence is 5.4 million [11,12].

Besides the health aspect, food poisonings also affect the economy due to the costs of hospitalisation, work absence, financial losses associated with consumers’ concerns of food quality, and the costs of legal proceedings [14]. The U.S. Department of Agriculture (USDA) estimated that the country spends 10–83 billion USD a year on aspects associated with food poisonings. In Australia, the corresponding cost is over a billion USD a year, and in New Zealand, it is 86 million USD [15].

## 2. Campylobacteriosis

The *Campylobacteriaceae* family is divided into four genera: *Campylobacter*, *Arcobacter*, *Dehalospirilum*, and *Sulfurospirilum* [26]. *Campylobacter* spp. are small (0.2–0.9 μm wide and 0.2–5.0 μm long), spirally curved, Gram-negative rods that do not form spores. They move in a way that resembles a corkscrew. This movement is possible due to a single, polar flagellum positioned on one or both ends of the cell [27,28]. Thirty-two species and 13 subspecies of those bacteria were identified. As pathogens, the greatest role is played by *Campylobacter jejuni* subs. *jejuni* (95% of cases of zoonoses) and *Campylobacter coli* (5% of infections) [26,29]. They differ from other pathogenic bacteria transmitted by food as they have the ability to grow in an atmosphere containing nearly 10% of CO_2_ and 5% of O_2_ (microaerophils) at a narrow range of temperatures ranging from 30 to 46 °C (the optimum growth temperature is 40–42 °C), which makes them thermophilic [30,31]. Growth of those microbes is not observed at water activity (a_w_) below 0.987, while the optimum value is 0.997. In conditions that do not favour growth, those bacteria are able to form viable but nonculturable cells (VBNC). Moreover, *Campylobacter jejuni* may survive for more than 4 h at 27 °C, which prevents these bacteria from multiplying outside animal hosts or in food during storage [28]. The other feature that allows survival of *Campylobacter* spp. in unfavourable conditions is their ability to form a biofilm on abiotic surfaces, which ensures a supply of nutrients and mechanical protection even though they cannot grow [32].

Already in 1909, bacteria belonging to genus *Campylobacter* were a known cause of animal diseases, but only as late as in 1980 the discovery was made that they also cause health problems in humans [33]. The incidence of infections caused by *Campylobacter* spp. has been constantly growing. Currently it is the most common foodborne bacterial zoonosis in the world [33,34]. It is estimated that *Campylobacter* spp. cause 500 million infections in the world every year [34]. In the European Union, the number of cases of campylobacteriosis has been the highest of all zoonoses since 2005, with the number of confirmed cases of infection that year being 197,363. After 2010, the number of people diagnosed with this disease has been over 200 thousand a year. In 2012, the EFSA noted 214,268 cases, and in 2015, the number of noted infections rose to 229,213, reaching 246,307 in 2016 [1,13,16,17,18,19,20,21,22,23,24,25]. It is estimated that in the United States, campylobacteriosis affects a million people a year, and in Canada, there are over 200 thousand cases registered each year [35,36]. Cases of campylobacteriosis have become common also in Africa, Asia, and the Middle East, particularly in children [32].

Sequencing of the *C. jejuni* NCTC 11168 genome demonstrated the existence of genes that code some proteins with infectious potential. Despite numerous studies on the molecular genetics of *Campylobacter* spp., their mechanisms of pathogenicity and virulence remain poorly understood [37]. Although the bacteria are considered to be susceptible to stress associated with environmental conditions, in the course of evolution, they were able to develop some complex mechanisms of survival and virulence, as presented in Table 1 [38,39].

In 1981, British doctor of medicine David A. Robinson determined that the human infective dose of *Campylobacter jejuni* is at the level of 500 to 800 microorganisms. The dose was later confirmed in other studies [41]. In 1988, doctor Robert E. Black and colleagues carried out a study on 111 adult volunteers in Baltimore. The subjects were administered 150 mL of pasteurised milk inoculated with two different strains of *Campylobacter jejuni* isolated during the outbreak of campylobacteriosis in Connecticut and Minnesota. The infective dose ranged between 8 × 10^2^ and 2 × 10^9^ bacteria. After administration, volunteers were followed up by physicians for 12 days, and samples of stool were collected during that period. The study confirmed that a low infective dose such as 800 *Campylobacter* bacteria is sufficient to cause the disease, and the risk of infection increased with increasing inoculum. However, the severity of symptoms was not dependent on the bacterial count [50]. There are also claims that 360 colony-forming units (CFU) of *Campylobacter* spp. could cause symptoms associated with campylobacteriosis, and 9 × 10^4^ bacteria is considered the optimum infective dose [27]. The disease incubation time usually ranges between 1 to 7 days before the development of symptoms and is longer in the case of individuals exposed to a lower infective dose. Symptoms accompanying the infection range from watery diarrhea to bloody stool, with fever, abdominal pain, vomiting, and dehydration. Symptoms disappear within 5–7 days [27,51]. In developed countries, the course of the disease is usually more severe compared with developing countries [33]. Moreover, campylobacteriosis may be associated with complications occurring in 1% of cases. Possible complications include: peripheral neuropathies, including the Guillain–Barré Syndrome (GBS, neurological disorder characterised by weakness of limbs, possible involvement of respiratory muscles, anaemia, and sensory loss); reactive arthritis (REA, involving knees and ankles, occurring about a month after infection and developing for as long as 5 years); and functional intestinal disorders, including irritable bowel syndrome (IBS) [27,46,52,53].

**Figure 3:**
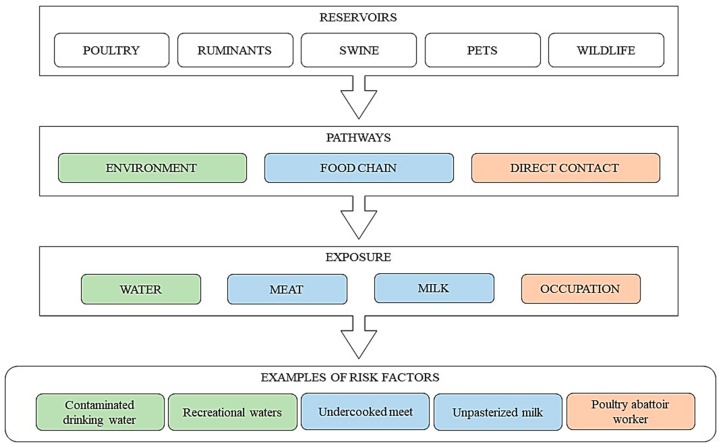
Reservoirs, transmission routs, and examples of source of infections caused by *Campylobacter* genus [56,66].

Campylobacteriosis is most often caused by the consumption of contaminated poultry, beef, or pork (Figure 3). It was determined that nearly 30% of all cases of infection were caused by the consumption of poultry, including 50–80% of isolated *Campylobacter* spp. strains of chicken origin, 20–30% of cases caused by pathogens from cattle, and a low percentage of pathogenic strains originating from other sources, including game [54,55]. Pathogenic bacteria which belong to *Campylobacter* genus do not proliferate outside the alimentary tract of warm-blooded animals but may survive as long as several weeks in food products, especially those stored at low temperatures [47,56].

Poultry consists of broilers, hens, turkeys, ducks, and ostriches, of which the meat industry uses mostly broiler chickens (*Gallus gallus*) [41,46]. *Campylobacter* spp. colonise the mucosa of the caecum and cloaca crypts of infected chickens, but may also be present in the spleen, blood, and liver [57]. The bacterial count per 1 gram of chicken faeces may reach the level of 10^10^, causing no infection and leading to no changes in caecal mucosa [40,57]. In newborn chickens before 3rd week of life, no presence of *Campylobacter* is found, which may be associated with the presence of antibodies from the maternal organism, the addition of antibiotics in feed, and development of the intestine and its microbiota [57,58]. After that time, if a single bird in the flock contracts the infection, it will be transmitted to the rest within days (approximately 3 days) through pathogen-containing faeces, or by rodents, water, insects, or farm workers [57,58,59]. It is estimated that in the EU, the amount of chicken meat available in retail markets containing *Campylobacter* spp. ranges between 60% and 80%, whereas in United States the amount is up to 98% [41].

Ruminants, including cattle, sheep, and goats, also act as a reservoir for *Campylobacter* bacteria [41,56]. The alimentary tract of cattle is colonised by *Campylobacter* spp. mostly in the gut (duodenum, jejunum, small and large intestines), rather than in the rumen [41,60]. It is estimated that these bacteria are present in approximately 80% of animals in an infected herd. The bacteria are less easily transmitted among sheep. The ratio of infected animals in a herd is estimated at 20% [41]. Although there are no studies on *Campylobacter* infections in small ruminants, there are some data regarding pathogens isolated from sheep carcasses and from lamb available in retail markets, as well from the liver, gallbladder, intestinal content, and faeces [61]. In the case of ruminants, the presence of *Campylobacter* bacteria in their alimentary tract is usually asymptomatic but may account for miscarriages in cows and sheep [56]. Besides the intestine, *Campylobacter* spp. may be present on the surface of hooves, in bristles, or in lymphatic nodes [47]. Not only meat products may be a threat but also dairy [41,56]. Raw milk is most often cross-infected with *Campylobacter* spp. during milking or as a result of udder infection [28]. Despite that broad spectrum of food products obtained from that group of animals, the most common source of infection transferred from ruminants is the environment, namely surface water, soil, air, pets (particularly cats and dogs), wild animals, and livestock serving as infection vectors [47,56].

*Campylobacter* spp. also inhabit the alimentary tract of 38–63% of pigs, but infections resulting from the consumption of pork are rare (0.4% of all confirmed cases of campylobacteriosis [56]. Pigs are considered to be the main reservoir of *Campylobacter coli* (90% of strains isolated from pigs), contrary to other reported groups of animals that are mostly infected by *Campylobacter jejuni* [56,62]. Pathogenic *Campylobacter coli* demonstrate a higher resistance to commonly used antibiotics, such as macrolides or quinolons, compared to *Campylobacter jejuni* [63]. On the other hand, the species is not resistant to freezing and drying. For that reason, despite a high ratio of *C. coli* infected pigs in slaughterhouses, the pathogen is rarely isolated from porcine carcasses. *Campylobacter* genus bacteria are isolated from various porcine products, including hamburgers, roasted pork, and sausage [64]. The source of campylobacteriosis may also be bone pork (e.g., loin) and offal (the liver, heart, kidneys, and guts) [65].

Not only animals and food products of animal origin constitute a source of campylobacteriosis, but vegetables are also a frequent vector of transmission. Contamination of vegetables may be the result of direct or indirect contact with livestock faeces. *Campylobacter* spp. isolated from vegetables and fruit may remain on their surface for 1 to as many as 8 days [56]. Infection is rarely primary (in the field, as a result of fertilizing with slurry or use of contaminated irrigation water) but often secondary—in kitchens (both home and commercial). In order to ensure appropriate hygienic conditions, vegetables have to be carefully washed before being peeled [47,56].

## 3. Salmonellosis

Bacteria belonging to the *Salmonella* genus were named after doctor of veterinary medicine Daniel Elmer Salmon, who along with his assistant, Mr. Theobald Smith, in the process of searching for causes of cholera popular in hogs, isolated in 1885 a new species of bacteria—*Bacillus cholerasuis*, renamed to *Salmonella enterica* serovar Cholerasuis [67,68]. Those bacteria are Gram-negative, relatively anaerobic, nonsporulating, straight rods belonging to the *Enterobateriaceae* family [67,69,70]. They are intracellular facultative pathogens, the size of which ranges between 2 and 3 μm. The shape of the rod is maintained due to the bacterial cytoskeleton made of an actin-resembling protein [71]. *Salmonella* spp. rods may survive in variable conditions. They are able to grow at temperatures ranging between 8 and 45 °C (optimum temperature 37 °C), at the pH of the environment from 4.0 to 9.5 (optimum pH 6.5–7.0), and in conditions of low water activity of 0.94 [68,72]. Those bacteria pose a great threat to the food industry because they are able to adapt to environmental conditions that are significantly different from their normal range of growth. Some strains are able to grow at 54 °C, and others even at 2–4 °C [68]. Pathogenic *Salmonella* spp. can move using the peritrichal flagellum. The bacteria are also able to ferment lactose, producing bisulfates. They are oxidase-negative and catalase-positive. *Salmonella* spp. may also be identified based on their biochemical properties of urease hydrolysis and the ability to grow in the presence of citrate as the sole source of carbon [73,74].

The nomenclature of the *Salmonella* genus is complex and inconsistent in the aspect of dividing this bacteria genus into species, subspecies, subgenera, groups, subgroups, and serotypes (serovars). The *Salmonella* genus is now divided into two species: *S. bongori* and *S. enterica*, based on genomic relatedness and biochemical reactions [75]. In 2005, a third species was classified, *S. subterranea*, but shortly after that it was shown not to belong to *Salmonella* genus [76]. The *Salmonella* genus is divided into seven subspecies: I, II, IIIa, IIIb, IV, V, and VI. This division is based on usual habitat, biochemical, and genetic criteria [75,77,78]. There are over 2500 serotypes. Each serotype has been identified according to differences in structure of the lipopolysaccharide O-antigen (somatic) and of the H-antigen (ciliary) [77,79]. The *S. bongori* species involves serotypes belonging to subspecies V, and other subspecies, including I, II, IIIa, IIIb, IV, and VI, belong to the *S. enterica* species. They are: *S. enterica* subsp*. Enterica*, *S. enterica* subsp. *Salamae*, *S. enterica* subsp. *Arizonae*, *S. enterica* subsp. *Diarizonae*, *S. enterica* subsp. *Hountanae*, and *S. enterica* subsp. *indica*, respectively [78,80]. Most serotypes are classified as *S. enterica* subsp. *enterica* (subspecies I) and they are responsible for 99% of salmonellosis cases in humans and warm-blooded animals. They are also often isolated from birds [78,81,82]. The ability to adapt to the conditions in the host organism and the resultant pathogenicity depend on the serotype of Salmonella genus bacteria. *S*. *typhi* and *S*. *paratyphi* A, B, and C are pathogenic for humans, but their presence in animals is asymptomatic. On the other hand, serotype *S*. *cholerasuis*, carried mostly by pigs, also causes salmonellosis in humans, and common serotypes *S*. *enteritidis* and *S*. *typhimurium* cause infections of the human gastrointestinal tract, as well as various symptoms in infected animals [83,84].

It is estimated that *Salmonella* spp. are the cause of over 90 million of diarrhea-associated diseases a year in the whole world, with 85% of those cases being linked to food [85]. The literature also reports the estimated annual number of cases of salmonellosis in the world, ranging between 200 million to over 1 billion [86,87]. The expected world fatality rate associated with salmonellosis is over 150 thousand. Fatalities are most often observed in children below the age of 4 years who are infected with serotypes Enteritidis or Typhimurium [80,88]. A reduced incidence of salmonellosis has been observed in the EU. The number of confirmed cases was 176,395 in 2005, 131,468 in 2008, 99,020 in 2010, 91,034 in 2012, 94,625 in 2015, and 94,530 in 2016, thus indicating a slightly increased incidence in recent years [1,13,16,17,18,19,20,21,22,23,24,25,80]. Over a million cases of salmonellosis a year are estimated in the United States. Nearly 20,000 require hospitalisation and there are approximately 400 cases of death resulting from infection with *Salmonella* [89,90].

The virulence of pathogenic *Salmonella* spp. is associated both with chromosomal and plasmid genes [91]. In the bacterial chromosome, there are large gene cassettes, called pathogenicity islands (SPIs), which code nearly 60 genes responsible for specific interactions with the host organism [91,92]. The *Salmonella* spp. infection cycle starts after the ingestion of microbes. Through the stomach, the bacteria reach the small intestine. The pathogenicity of Salmonella spp. depends on the serotype and the host’s immunity, and its virulence is determined by the factors presented in Table 2 [93,94].

Pathogenic bacteria which belong to the *Salmonella* genus cause three types of salmonellosis in humans: noninvasive and nontyphoid, invasive and nontyphoid, and typhoid fever caused by the serotype *S*. *typhi*, as well as paratyphoid fever caused by two serotypes *S*. *paratyphi* A, B, and C [82,103].

Serotypes causing typhoid fever are transmitted between people with no mediation of an animal as a vector [70]. Infection may be associated with food or water, and the presence of those bacteria is closely related to poor hygiene. Transmission is affected by overpopulation in areas of poor sanitary conditions [104]. Stating the exact number of typhoid fever cases is impossible. It is believed that the world incidence may reach as high as 21 million a year, with over 200,000 fatal cases [82,105]. The highest number of cases of typhoid fever is noted in Africa and in Southeast and Central Asia [73,106]. Typical symptoms include: headache, stomach ache, fever, diarrhea or constipation, and loss of appetite, but other possible symptoms are: respiratory problems, lethal neurological changes, perforation of the intestine, and hepatic and splenic injury [67,107].

Salmonellosis is caused by all nontyphoid serotypes of the *Salmonella* genus (excluding *S*. *typhi* and *S*. *Paratyphi* A, B and C), isolated both from humans and animals, including livestock [82]. Serotypes *S*. Typhimurium, *S*. *enteritidis*, *S*. *newport*, and *S*. *heidelberg* are most often responsible for food poisoning, but *S.* Cholerasuis and *S.* Dublin also cause diarrheic diseases [73,82]. Pathogens enter the organism with water or food infected with faecal microbiota. For that reason, the environment constitutes an important vector in the dissemination of *Salmonella* spp. [73,82,84]. The infective dose ranges between 10^6^ and 10^8^ cells, but in some people, even the dose of 10 cells may lead to the development of salmonellosis [108,109]. Incubation of the disease lasts for 6–72 h, depending on the infective dose and the host’s condition. In the majority of cases, the infection lasts no longer than 7 days [67,90,109]. Typical symptoms of infection with nontyphoid serotypes of *Salmonella* spp. are stomach ache and diarrhea, but other possible symptoms include: vomiting, nausea, fever, shivers, muscular or articular pain, cramps and loss of appetite [85,107,109,110]. After the disappearance of symptoms, *Salmonella* may still reside in the intestines of an adult for 4 weeks, and in children for up to 7 weeks. A small number of people demonstrates an asymptomatic carrier state of pathogenic bacteria for a year after the disappearance of symptoms [111]. In the majority of cases, nontyphoid salmonellosis does not require hospitalisation, and only fluid therapy is necessary in the cases associated with severe diarrhea. Antibiotic therapy is avoided in order to hinder the formation of drug-resistant strains, but in the case of a complication with bacteraemia, antibiotic therapy with fluoroquinolons or 3rd generation cephalosporins is necessary [82,85,112]. Bacteraemia develops in 5–10% of people infected with *Salmonella* spp. and may lead to focal infections, such as meningitis, endocarditis, arthritis, and osteitis [77,110].

Infection with *Salmonella* may be a result of direct contact with infected animals or indirect contact via their environment. Also, the consumption of infected products or food prepared in an infected environment may account for food poisoning [89]. The main source of pathogenic *Salmonella* causing food poisoning in humans is eggs and egg products [113]. Also, pigs and pork constitute an important reservoir for numerous serotypes of those pathogens, as well as cattle and dairy products (Table 3) [113,114,115,116]. Most often, meat becomes infected with *Salmonella* spp. during the production process when the bacteria that are abundant in animal intestines may become transferred onto meat as a result of careless processing or improper hygiene [117]. Fresh meat is a good environment for the growth of pathogenic *Salmonella* spp. due to a high content of nutrients, pH of 5.5–6.5, and high water activity (aw = 0.98–0.99) [118]. Also, vegetables contaminated with animal faecal microbiota may constitute a reservoir for *Salmonella* spp. [119].

The intestines of poultry, especially of chicken and turkeys, are asymptomatically colonised by *Salmonella* spp. as a result of a horizontal or vertical transmission of bacteria at the stage of primary production [108,120]. The horizontal route of infection includes contaminated feed and water, as well as bedding, soil, air, and farm personnel [121,122]. The vertical route includes direct infection of offspring by its flock [116,121]. *Salmonella* spp. may be present in as much as 65% of individuals in a flock. Serotypes colonising the gastrointestinal system of poultry are variable, depending on the geographic location and the time of the year, and part of them are repeatable (e.g., *S*. Enteritidis, *S*. Typhimurium) [121,123,124]. Besides the unrestricted dissemination and colonisation of intestines, *Salmonella* spp. may also be transferred to the liver, spleen, and ovaries [120]. *S*. Gallinarum and *S*. Pullorum serotypes are pathogenic for poultry but not for humans. However, they cause significant losses in the poultry industry [73,125]. Many strains isolated from poultry and responsible for food poisoning in humans demonstrate resistance to selected antibiotics. For that reason, much attention is drawn to eggs and poultry meat as sources of *Salmonella* bacteria [124]. Infected birds are the primary source of *Salmonella* spp. infection in the production environment. Bacteria may be introduced at every stage of the production process and cause infection of the final product [121,126]. In the case of egg production, egg content may be infected at the stage of forming because of bacterial colonisation of the hen’s genital system. Moreover, eggs may be also infected through the environment, and pathogenic bacteria may be present not only on the surface of an egg shell, but also penetrate the inside [87].

Pigs and pork also constitute an important reservoir of *Salmonella* spp. Infection with these pathogens is asymptomatic in those animals and affects neither growth nor production parameters. The only exception is *S*. Cholerasuis, which makes pigs ill [72,127]. Pathogenic bacteria colonise the intestines of those animals, reaching the count of 10^3^ cfu/g, and in animals subject to stress-inducing conditions (e.g., a transfer to a slaughterhouse), the count may be increased, which may lead to the development of the symptoms of infection and increased cross-infections [127,128]. Pork becomes contaminated with *Salmonella* spp. in slaughterhouses by direct or indirect contact with a carcass with the intestinal content or faeces, or through the production environment [115]. Pigs may be infected at the stage of breeding, via feed, infected by animals from other farms, from the environment, or by vectors such as insects, fleas, birds, dogs, or cats [129].

*Salmonella* spp. is also increasingly present in cattle, both bred for meat and for milk [130]. Almost 40% of a herd may be infected, and the risk of infection increases with the size of the herd [131]. Beef is usually infected at the stage of slaughter, and pathogenic *Salmonella* bacteria are introduced to the breeding environment with externally sourced animals, water, feed, rodents, birds, and the farm personnel [132]. Cattle may be asymptomatic carriers of the pathogenic bacteria (the bacteria may remain in their gastrointestinal tract for periods ranging between a few months and a year), but there may also be symptoms of infection present, including diarrhea, fever lasting for up to 7 days, anorexia, dehydration, reduced milk production, miscarriages, or the presence of toxins in blood [131,132]. Mortality associated with *Salmonella* spp. infections is high in calves but still not very common, just like miscarriages. Salmonellosis in cattle puts producers at risk of direct economic loss associated with mortality or body weight loss, and of indirect loss caused by reduced feed conversion or medical care costs [131].

## 4. Yersiniosis

There are 17 species in the *Yersinia* genus belonging to the *Enterobacteriaceae* family. Three of them are pathogenic for humans—*Y. pestis* (transmitted by fleas and air bacteria causing plague), *Y. pseudotuberculosis* (the aetiologic factor of rodentiosis), and *Y. enterocolitica* (the aetiological factor of yersiniosis)—and cause diseases of the gastrointestinal tract in humans. Infections associated with *Y. pseudotuberculosis* are rather rare [133,134,135].

The bacteria belonging to the *Yersiniae* genus are rods or cocci, 0.5–0.8 μm wide and 1–3 μm long, nonsporulating. Those microbes are mobile in temperatures ranging between 22 and 30 °C due to a flagellum positioned on a pole of the bacterial surface. They are not mobile at 37 °C [136]. *Y. enterocolitica*, causing the zoonosis called yersiniosis, are Gram-negative, relatively anaerobic, catalase-positive, nonsporulating, and absolutely psychrophilic enteropathogens [133,135]. Those bacteria are able to grow at temperatures ranging between 0 and 45 °C, whereas their optimum growth temperatures range between 25 and 32 °C [133,137]. *Y. enterocolitica* are able to survive in the VNBC condition and proliferate and produce a thermostable toxin in cooling conditions (4–8 °C). Because of that, they pose a great problem for food [138,139]. These pathogens grow at an environmental pH lower than 9, and the water activity must not be lower than 0.96. Low pH and the presence of organic acids, including acetic acid, lactic acid, and citric acid, may inhibit the growth of *Y. enterocolitica* [140].

There are six different biotypes of *Y. enterocolitica* reported in the available sources, namely, 1A, 1B, 2, 3, 4, and 5, classified based on biochemical reactions: production of indole, production of acids from xylose, trehalose, saccharose, sorbose and sorbitol, hydrolysis of salicin and eskulin, dekarboxylation of ornitine and o-nitrophenyl-β-d-galactopiranoside, Voges-Proskauer reaction, and reduction of nitrates [141,142]. Eleven of the serotypes belonging to those biotypes were associated with the development of yersiniosis in humans. The highest incidence of yersiniosis in Europe is caused by the bacteria belonging to biotype 4, serotype O:3 [142]. Biotype 1A is common in the environment, and considering the absence of the majority of virulence markers (chromosomal and plasmid pYV), it is considered nonpathogenic [142,143,144]. Serotypes of *Y. enterocolitica* are classified based on the structure of the lipopolysaccharide O-antigen, constituting a part of the superficial lipopolysaccharide layer of the cell [145]. The most pathogenic bioserotypes/serotypes are: 1B/O:8, 2/O:5,27, 2/O:9, 3/O:3, and 4/O:3, with the last one being responsible for the highest incidence of yersiniosis among humans in Europe, Canada, Japan, and China [146,147].

Virulence factors of *Y. enterocolitica* are pieces of genetic information contained in the chromosome (more stable virulence markers) and in the plasmid of Yersinia—pYV, approximately 70 kilo base pair (kb) long [140,148]. After consumption of infected food or water, *Y. enterocolitica* reach the distal part of the small intestine and the proximal part of the large intestine, start to proliferate there, and colonise the environment, leading to infection of the host organism [140,141,149]. Mechanisms of virulence of *Y. enterocolitica* are presented in Table 4.

The infective dose of pathogenic *Y. enterocolitica* is higher compared to that of other pathogenic bacteria present in food, and is 10^8^–10^9^ cells [164]. Incubation time is nearly 3–7 days, but may range between 1 and 11 days [133,166]. Symptoms of the disease may be mild but may also have a form of severe gastritis and enteritis, disappearing within 1–3 weeks [137,141,166]. Yersiniosis affects all humans, but children under the age of 5 years, people with reduced immunity, and the elderly are more at risk. The disease is manifested by fever, stomach ache, and diarrhea (often bloody), and in adult patients often resembles appendicitis [140,146,167]. Complications may involve erythema nodosum, osteoarthritis, bacteraemia, purulent hepatitis, splenitis, or nephritis, myocarditis, and less often sepsis and endocarditis [137,141,151]. Infection with *Y. enterocolitica* is often associated with nonspecific symptoms, resembling diseases of other aetiology. For that reason, many cases of yersiniosis are misdiagnosed [167]. In the case of infected individuals with reduced immunity, antibiotic therapy is recommended. According to WHO recommendations, the therapy is based on tetracyclines, chloramphenicol, gentamycin, or cortimoxazole [149]. Due to mutations occurring in the bacterial chromosome, the phenomenon of antibiotic resistance to fluoroquinolons is observed in *Y. enterocolitica*, but also to ampicillin, ticarcilin, amoxicillin/clavulanian, cefazolin, and cefalotin [168].

According to US Centers for Disease Control and Prevention (CDC) estimates presented in 2016, yersiniosis affects nearly 117,000 people a year in the United States, including 640 cases requiring hospitalisation and 35 resulting in death [169]. In the EU, a decreasing tendency of confirmed cases of yersiniosis has been observed between 2005 and 2016. The incidence in 2005 was 9630, and in 2016 was 6861 [1,13,16,17,18,19,20,21,22,23,24,25,166,170]. Incidence of *Y. enterocolitica* infections is carefully monitored in developed countries, but no sufficient diagnostics are available in Africa and the Middle East, and no exact number of cases of the disease is known for those areas [171].

The main source of yersiniosis in humans is food, in particular raw or undercooked pork, but also fresh and pasteurised milk and other dairy products, infected plants, seafood, and drinking water [167,172]. Food may be contaminated primarily or by contact with an infected surface or equipment [167]. Although pigs are a leading reservoir of *Y. enterocolitica*, those bacteria are abundant in the environment and are also isolated from other animals—including poultry, cattle, sheep, and goats—and from wild animals such as rodents, deer, boars, and also cats and dogs [135,173]. Pigs carrying *Y. enterocolitica* do not have any symptoms of infection. Pathogens occupy their tongues, oral cavities, tonsils, lymph nodes, and intestines and are present in their faeces [135,174,175]. During the slaughter and processing of meat, *Y. enterocolitica* may be transferred from infected tissues onto other meat. Meat from the areas close to the head and sternum is the most exposed [176].

## 5. Listeriosis

The aetiological factor of listeriosis in humans is the bacteria *Listeria monocytogenes*. They were first described in 1926 by Murray and colleagues during an outbreak involving rabbits and guinea pigs in a laboratory in Cambridge (Great Britain). At that time, the bacteria were called *Bacterium monocytogenes* [177,178]. In the early 1980s, this pathogen became associated with human food poisonings [179,180].

The *Listeria* genus incorporates 17 bacteria species divided into two groups: *Listeria* sensu stricto, which includes *L. monocytogenes*, *L. seelgeri, L. ivanovii, L.marthii, L. welshimeri*, and *L. innocua*; and *Listeria* sensu lato, which consists of another 11 species divided into three clades, among which there are five new species (*L. aquatic, L. floridensis, L. cornellensis, L. grandensis,* and *L. riparia*) described by den Bakker et al. (2014). Only *L. monocytogenes* are considered pathogenic for humans [177,181,182]. The bacteria are Gram-positive, relatively anaerobic, oxidase-negative, and catalase-positive organisms having the form of small mobile rods (0.4–0.5 × 1–2 μm) that are unable to produce spores [177,183]. *L. monocytogenes* are mobile in temperatures ranging between 22 and 30 °C, but at 37 °C they do not form a flagellum [183,184]. Those bacteria are able to grow in unfavourable conditions, such as high salt concentration, low temperature (growth already at −0.4 °C), and high temperature (maximum 45 °C), and also demonstrate growth over a broad spectrum of pH (from 4.4 to 9.4) and in a low water activity of 0.9 [178,180,185]. Total inactivation of those pathogenic bacteria occurs at 75 °C [186].

Thirteen serotypes of *L. monocytogenes* were identified (1/2a, 1/2b, 1/2c, 3a, 3b, 3c, 4a, 4b, 4c, 4d, 4e, 4ab, and 7). The following are the serotypes that are most commonly isolated from infected humans: 1/2a, 1/2b, 1/2c, and 4b [186,187]. Strains belonging to separate serotypes differ in terms of the antigenic determinants located on the cellular surface, including that of the thermostable somatic antigen O and the thermophilic ciliary antigen H [186,188]. Based on the differences in the nucleotide sequence of three genes (*flaA, iap,* and *hly*), four evolution lineages are also classified (I, II, III, and IV). Individual serotypes are affiliated with those lines [186,187,188].

*L. monocytogenes* are intracellular pathogens, which means that for infection of the host organism, bacteria have to not only penetrate intestinal cells, but also cells of the host’s spleen, liver, brain, heart, and placenta [189,190]. Following the invasion of cells, pathogenic bacteria are located in the vacuole. After leaving that location, bacteria start proliferating, and due to an actin-based motility mechanism, they may travel to other cells without triggering the host’s immunological response [191,192]. The pathogenicity of those bacteria is based on the production of the following virulence factors, listed in Table 5.

Listeriosis much more often affects elderly people and pregnant women, as well as those individuals whose immunity is weak for various reasons, including neonates and children under the age of 5 years, organ transplantation patients, cancer patients, and HIV carriers [200,207]. Infections associated with *L. monocytogenes* are characterised by a long incubation period ranging between 1 and as many as 70 days (average length of 8 days). The period may be even longer in pregnant women (17–67 days). A different incubation time is also noted in individuals with the symptoms of bacteraemia (1–10 days; average: 2 days), feverish gastroenteritis (mean: 24 h; range from 6 h to 10 days), as well as in patients with listeriosis attacking the nervous system (1–14 days, average: 9 days) [208,209]. The exact infective dose of *L. monocytogenes* is not defined because it depends on the susceptibility of the host organism and the virulence of a particular strain. It is agreed that 10^6^ cfu/g is sufficient to trigger symptoms [209]. Symptoms of listeriosis may appear within 24 h and in otherwise healthy adults they may be: articular pain, headache and stomach ache, diarrhea, nausea, vomiting, lack of appetite, weariness, and somnolence. They usually disappear within 1–3 days [180,184]. In the case of pregnant women, infection with *L. monocytogenes*, besides fever and diarrhea, may lead to miscarriage. In neonates, the infection may lead to sepsis, pneumonia, or meningitis [207]. Ampicillin and amoxycyclin are most often used in treatments of listeriosis, sometimes with gentamycin, erythromycin, and vancomycin [184,190]. *L. monocytogenes* bacteria are also susceptible to penicillin, rifampicin, and cotrimoxazol and demonstrate natural resistance to fluoroquinolons and cephalosporins [210].

Although listeriosis is not a common disease, the majority of its cases are associated with necessary hospitalisation. The disease is also associated with a high mortality rate, reaching 20–30%, and for risk-group patients even 75% [211,212]. The number of confirmed cases of listeriosis among the inhabitants of the EU demonstrated a growing tendency. In 2005 it was 1439 and in 2016 reached 2530 cases [1,13,16,17,18,19,20,21,22,23,24,25]. The CDC estimates that there are 1600 cases of listeriosis annually in the United States, and approximately 260 cases of associated death [213]. The Australian official organization, the Food Standards, reports 150 cases of patients with listeriosis hospitalised every year, including 10% dying because of the infection [214].

*L. monocytogenes* are common in the environment and isolated from the soil, surface water, waste water, faeces, feed, agricultural environments, and food processing plants [215]. Moreover, pathogenic strains of those bacteria may also colonise domestic animals, such as cattle, sheep, goats, horses, poultry, but also wild birds, fish, and shellfish [216]. It is estimated that 99% of the cases of listeriosis are associated with contaminated food, although the long incubation period largely hinders clear determination of the infection source [209,217]. Pathogenic *L. monocytogenes* are often isolated from food products for direct consumption, including meat (beef, turkey, hotdogs, cooked ham, pork jelly), milk, dairy (made of pasteurised milk: butter, soft cheese, cottage cheese; made of fresh milk: Mexican style soft cheese, home-made cheese), fish (smoked, marinated, carpaccio), and other seafood (crab, shrimps, smoked mussels), but also ice cream, fresh vegetables (corn, celery, cabbage) and fruit, e.g., cantaloupe [178,209,216,217].

*L. monocytogenes* causes infection both in wild and domestic ruminants, as well as in monogastric animals. Dissemination of pathogenic bacteria among animals is most often a result of poor quality feed [218]. Both in humans and animals, listeriosis may be associated with symptoms including enterogastritis, sepsis, and infections of the CNS (meningitis or myelitis), and also may lead to miscarriage [219].

Ruminants may be asymptomatic carriers of *L. monocytogenes*, but there are also cases of infective diseases caused by those pathogenic bacteria. In both cases, animals disseminate bacteria in the environment with faeces. In favourable conditions, microorganisms are able to survive there for a long period of time. That may account for infection of other animals in the herd, as well as of animals in other herds using the same pasture [113,220]. In the case of outbreaks among cattle, sheep, and goats, the bacteria colonise 7.5–29.4% of individuals in the herd (in Europe), but despite the low ratio of infected animals, their mortality may reach 100% [221,222]. *L. monocytogenes* may infect cattle of both sexes and various ages, but animals below the age of 3 years are more susceptible. In the case of sheep, the course of the disease is more severe than in cattle. In that latter case, the ratio of recovery may reach 50% [223]. Even though there is evidence suggesting beef, raw milk, and milk derived products are sources of *L. monocytogenes* and the disease is defined as zoonosis, a direct link between ruminant and human listeriosis is not understood [222,224,225].

Pigs may also be carriers of *L. monocytogenes* and the infection may be asymptomatic. However, a neural form of the infection is also observed (in older animals) or sepsis (rarely). No pathogens are isolated from faeces of those animals [220,223]. Pigs may be infected via the gastrointestinal tract, but also via the respiratory tract and the conjunctiva [223]. Despite swine being mostly healthy carriers of *L. monocytogenes*, pork meat and derived products are linked to human infections and production-environment contamination [220].

Also, poultry may be colonised by *L. monocytogenes*. However, the consumption of infected poultry meat is not associated with a high incidence of listeriosis. There are also no data on the frequency of occurrence of those pathogens in poultry and meat obtained from them [211].

## 6. Conclusions

Livestock diseases are associated not only with the economic loss caused by reduced populations, reduced productivity, and veterinary care costs, but also pose a threat to human health [226]. Because of the increased demand for food related to the growing human population, we have become exposed to pathogens present in the production environment and food poisonings associated with them [2].

The best solution to limit the incidence of zoonoses in humans is to control and prevent dissemination of pathogens in animals constituting the main reservoir of the infections [227]. In industrialised countries, animals are examined before slaughter, there are vaccinations and monitoring of animals, and also the hygienic conditions of the production facilities are controlled (physical decontamination). However, those methods often prove ineffective in developing countries [226,227]. For these reasons, it is crucial that education in areas such as microbiology, sanitation, hygiene, food science, good agricultural and good manufacturing practices, and also implementation of risk assessment through hazard analysis and critical control points should be considered as necessary [228].

## Figures and Tables

**Table 1:** Attributes of *Campylobacter* genus bacteria allowing them to infect and survive in a host organism.

The Mechanism of Survival/Virulence	Description	References
Mobility	- moving against the persitalsis, reaching target sites in the intestine ^1^; - adhesion to host’s cells, formation of a biofilm, secretion of invasive proteins ^1^; - required flagella and a chemosensory system (regulation of the flagellar movement depending on environmental conditions) ^2^.	[40,41,42,43]
Drug resistance	- increasing antibiotic resistance resulting from the misuse of antibiotics in medicine, veterinary medicine, and agriculture ^2^; - acquiring antibiotic resistance while dwelling in the alimentary tract of livestock and humans ^2^; - resistance to fluoroquinolons (e.g., ciprofloxacin), macrolides (e.g., erythromycin), aminoglicosides (e.g., gentamycin, canamycin and streptomycin), tetracyclines, and β-lactames (e.g., penicillins and cephalosporins) ^2^.	[27,40,44,45,46,47]
Adherence to host’s epithelial cells	- initial colonisation of intestinal epithelium ^1^; - mediation of the adhesins on the surface of bacterial cells, including: CadF (an external membrane protein), PEB1 (periplasmatic binding protein), JlpA (lipoproteins engaged in adhesion to Hep-2 cells), and CapA (Camplyobacter A adhesion protein) ^2^.	[40,41,48]
Invasion of host’s cells	- avoiding immunological response ^2^; - significant role played by the external lipopolysaccharide bacterial core ^2^.	[39,41]
Production of toxins—cytolethal distending toxin (CDT)	- a protein composed of the subunits coded by genes *cdtA, cdtB*, and *cdtC* ^2^; - *cdtB* encodes the enzymatic part of the toxin ^2^; - *cdtA* and *cdtC* encode subunits responsible for binding the toxin to the membrane of an eukaryotic cell ^2^; - subunits CdtA, CdtB, and CdtC necessary for correct function of the toxin ^2^; - halting the eukaryotic cell during the G2/M phase of the cellular cycle, stopping from transition into the phase of mitosis—cellular death ^2^; - not all strains produce CDT ^2^.	[28,40,49]

^1^ Attributes common for *C. jejuni*; ^2^ Attributes common for *Campylobacter* genus.

**Table 2:** Factors determining the virulence of *Salmonella* genus bacteria.

The Mechanism of Virulence	Description	References
Adherence to host’s cells	Adherence modulated by: - fimbriae (protoplasmic outgrowths)—proteins interacting with the host’s receptors on the tips; - adhesins (proteins): BapA, SiiE, ShdA, MisL, and SadA; - flagellae (up to 10 randomly distributed over the cell surface)—mobility of the cell may indirectly facilitate adhesion.	[78,84,93]
Invasion and replication inside host’s cells	- after binding the pathogen to host’s cells; - transmission of effectors to the cytosol of the infected cell. Effectors stimulate the cell’s signalling system through the type III secretion. - secretion system coding genes are localised on SPIs; - effector proteins responsible for invasion and replication of *Salmonella* spp. influence also the survival and stimulated production of proinflammatory cytokines (development of infection).	[86,93,95,96,97]
Polysaccharide coating	- the superficial part of the membrane bilayer of Gram-negative bacteria is composed almost entirely of lipopolysaccharides (LPS); - Lipid A—the lipid part of the external lipopolysaccharide layer, causes various immunological responses of the host organism (e.g., activation of the complex of toll-like receptors 4-MD2-CD14, which leads to expression of proinflammatory molecules or adhesion proteins).	[98,99,100,101,102]
Production of toxins	- endotoxins (lipid A); - exotoxins (cytotoxins and enterotoxins).	[99,102]

**Table 3:** *Salmonella* spp. serotypes most often isolated from animal reservoirs in European Union countries. Underlined serovar was the one isolated most frequently [1,13,25].

Year	Animal Reservoirs			
	Broiler	Cattle	Pigs	Turkey
	*Salmonella* Serotypes			
2014	*S*. Typhimurium *S.* Infantis *S*. Enteritidis *S.* Mbandaka *S*. Livingstone *S*. Kedougou *S*. Enftenberg *S*. Kentucky *S*. Typhimurium Copenhagen *S*. Brandenburg	*S*. Typhimurium *S*. Enteritidis *S*. Dublin *S*. Mbandaka *S*. Coeln *S*. Give *S*. Montevideo *S*. Anatum *S*. Bredeney *S*. Typhimurium Copenhagen	*S*. Typhimurium *S*. Infantis *S*. Derby *S*. Typhimurium monophasic *S*. Typhimurium Copenhagen *S*. Rissen *S*. London *S*. Muenchen *S*. Livingstone ar14	*S*. Typhimurium *S*. Infantis *S*. Derby *S*. Enteritidis *S*. Newport *S*. Hadar *S*. Stanley *S*. Saintpaul *S.* Virchow *S*. Kottbus
2015	*S*. Typhimurium *S*. Infantis *S*. Enteritidis *S*. Derby *S*. Typhimurium monophasic *S*. Livingstone *S*. Mbandaka *S*. Cerro *S*. Thompson *S*. Kedougou	*S*. Typhimurium *S*. Infantis *S*. Enteritidis *S*. Dublin *S*. Typhimurium monophasic *S*. Mbandaka *S*. Newport *S*. Goldcoast *S*. Brandenburg	*S.* Typhimurium *S*. Infantis *S*. Enteritidis *S*. Derby *S*. Typhimurium monophasic *S*. Goldcoast *S*. Rissen *S*. Brandenburg *S*. London	*S*. Typhimurium *S*. Infantis *S*. Enteritids *S*. Derby *S.* Typhimurium monophasic *S*. Newport *S*. Kedougou *S*. Branderburg
2016	*S*. Enteritidis *S*. Typhimurium *S*. Typhimurium monophasic *S.* Infantis *S*. Derby	*S*. Enteritidis *S*. Typhimurium *S*. Typhimurium monophasic *S*. Infantis *S*. Derby	*S*. Enteritidis *S*. Typhimurium *S*. Typhimurium monophasic *S*. Infantis *S*. Derby	*S*. Enteritidis *S*. Typhimurium *S*. Typhimurium monophasic *S*. Infantis *S*. Derby

**Table 4:** Mechanisms and factors facilitating infection of the host’s organism with *Y. enterocolitica*.

Virulence Mechanisms/Factors	Description	References
Adhesion and invasion of host’s cells	Adherence modulated by: - autotransporters (type V secretion systems), including YepE, YadA (superficial protein, coded by plasmid pYV, binding pathogens to epithelial cells, phagocytes, and components of cellular matrix, protection against neutrophils, autoaggregation of microbes, triggering the immunological reaction); - invasine (Inv)—encoded by the *inv* gene in the chromosome, expressed at temperatures up to 25 °C, at pH = 5.5. The gene may be expressed at 37 °C. It is present on the external bacterial membrane and participates in adhesion and invasion of intestinal epithelial cells (binding to β1-integrins on surface of M cells of Peyerąs patches); - fimbrial adhesins; - Ail protein—an extracellular membraneous protein encoded by the chromosomal gene *ail*, supports the YadA protein function, allows penetration of the rods into host’s cells, increases bacterial resistance to the bactericidal effect of the complement.	[136,138,150,151,152,153,154,155,156]
Secretion system type III	- encoded by genes localised on the pYV plasmid, expression at 37 °C with low concentration of calcium ions; - composed of effector proteins Yop (Yersinia outer proteins), proteins forming a needle YscF (Yersinia secretion protein F), translocator YopD, and the LcrV protein providing a shield; - YopB connects YopD with LcrV forming a membranous complex—transmission of effectors into the cells of the infected organism; - disturbs the function of both congenital and acquired immunological response, inhibits phagocytosis.	[137,138,149,157,158,159]
Mobility	- dependent on the presence of a flagellum; - necessary for the invasion of host’s cells—migration and adhesion to the intestinal epithelium.	[136,160]
Lipopolysaccharides	- endotoxins (lipid A)—a strong activator of the immunological response; - antigen O—part of the superficial membrane, affects adhesion and invasion of pathogens into host’s cells.	[135,136]
Thermostable enterotoxin Yst	- a protein encoded by the chromosomal gene *yst*; - not denaturated during 20 min heating at 100 °C; - functionally and structurally similar to the toxins of other bacteria causing food poisoning; - biotype 1A (no virulence markers) possesses the *ystB* gene encoding the heat resistant YstB toxin that may be secreted at 37 °C; - product of expression of the *ystA* gene, present in the case of virulent biotypes.	[135,136,144,161,162,163,164]
Production of urease	- neutralisation of the environment, survival of pathogens in the gastrointestinal system.	[153,155]
Avoiding the host’s immunological response	- YadA proteins (combined with the antigen O) and Ail—avoiding proteins of the complement system present in plasma; - Yop—modulation (delay) of the host’s immunological response, stimulation of the production of anti-inflammatory cytokines (inhibition of the inflammation).	[153,155,165]

**Table 5:** Factors determining the pathogenicity of *Listeria monocytogenes*.

Virulence Factors	Description	References
The adhesive protein (LAP)	- molecular mass 104 kDa; - adherence of the bacteria to the intestinal epithelium; - expression at a low availability of oxygen and nutrients, at 37–42 °C; - secreted by SecA2 to the outside of the cell and bound to the bacteria surface in the case of pathogenic strains.	[193,194,195]
Internalines	- adhesion and invasion of host’s cells; - anchored in the cellular wall or covalently bound to peptidoglycan; - the most important role is played by internalin A (InlA) and internalin B (InlB)—they allow production of a biofilm; - InlA—binding to E-cadherin receptors on the surface of host cells (responsible for intercellular interferences)—local changes of the cytoskeleton, allow bacterial penetration of host cells; - InlB—recognition of hepatocyte growth receptors (Met, gC1qR and glycosoaminoglycans)—internalisation of host cells; - InlA and InlB encoded by the operon *inlAB* localised in the bacterial chromosome; - other, e.g., InlJ, InlH, InlF, or InlC—responsible mostly for adhesion of pathogens to host cells.	[190,196,197,198,199,200]
Listeriolysin O	- a toxin encoded by the *hly* gene localised on the Listeria pathogenicity island 1 (LPI-1); - binding to cholesterol molecules contained in membranes and oligomerisation, allows penetration of listeriolysin into the membrane, forming openings, and finally allows *L. monocytogenes* getting out of vacuoles; - avoiding macrophages and penetration into the host cell cytosol; - affects signal pathways in the cytosol—modulation of the course of infection.	[190,201,202,203]
Secretion systems	- a path depending on Sec (a classical system of secretion of proteins marked with a signal peptide on the N terminal in prokaryotes); - TAT pathway; - FPE (fimbrilin protein exporter); - FEA (flagellar export apparatus)—homologous to the secretion system type III; - the choline pathway; - the Esx-1/Wss system.	[191]
Phospholipases C PlcA and PlcB	- exiting host cells, transport to neighbouring cells; - support the effect of listeriolysin O; - products of genes *plcA, plcB*, respectively, located on LPI-1.	[189,201,204]
Superficial proteins ActaA	- activation of the actin polymerisation process around bacterial cells—formation of the actin tail allowing L. monocytogenes to move and penetrate other cells of the organism, - possible promotion of autoaggregation, formation of a biofilm, avoiding autophagia (survival of the pathogen in the intestine); - a product of expression of the gene *actA* localised on LPI-1.	[201,205,206]
The OrfX protein	- oxidation of macrophages—development of infection; - a product of expression of the gene *orfX* localised on LPI-1.	[192]
